# The Anorectic Effect of GLP-1 in Rats Is Nutrient Dependent

**DOI:** 10.1371/journal.pone.0051870

**Published:** 2012-12-17

**Authors:** Darleen Sandoval, Jason G. Barrera, Margaret A. Stefater, Stephanie Sisley, Stephen C. Woods, David D. D’Alessio, Randy J. Seeley

**Affiliations:** 1 Division of Endocrinology and Metabolism, University of Cincinnati, Cincinnati, Ohio, United States of America; 2 Department of Pyschiatry, University of Cincinnati, Cincinnati, Ohio, United States of America; University of Medicine & Dentistry of NJ - New Jersey Medical School, United States of America

## Abstract

GLP-1-induced insulin secretion from the β-cell is dependent upon glucose availability. The purpose of the current study was to determine whether CNS GLP-1 signaling is also glucose-dependent. We found that fasting blunted the ability of 3^rd^ cerebroventricularly (i3vt)-administered GLP-1 to reduce food intake. However, fasted animals maintained the anorexic response to melanotan II, a melanocortin receptor agonist, indicating a specific effect of fasting on GLP-1 action. We also found that i3vt administration of leptin, which is also decreased with fasting, was not able to potentiate GLP-1 action in fasted animals. However, we did find that CNS glucose sensing is important in GLP-1 action. Specifically, we found that i3vt injection of 2DG, a drug that blocks cellular glucose utilization, and AICAR which activates AMPK, both blocked GLP-1-induced reductions in food intake. To examine the role of glucokinase, an important CNS glucose sensor, we studied glucokinase-heterozygous knockout mice, but found that they responded normally to peripherally administered GLP-1 and exendin-4. Interestingly, oral, but not i3vt or IP glucose potentiated GLP-1′s anorectic action. Thus, CNS and peripheral fuel sensing are both important in GLP-1-induced reductions in food intake.

## Introduction

Over 60 years ago, Jean Mayer proposed that peripheral blood glucose fluctuations were detected by specific neurons within the CNS and that this information was used as a signal to regulate food intake [1]. Since then, several studies have found that depriving the CNS of glucose using pharmacological blockade of glucose metabolism stimulates food intake[2]–[4] while acute [5] and chronic [6] intracerebroventricular (i3vt) and peripheral glucose administration [7] suppresses food intake in rats. Acute intravenously-induced hyperglycemia is also associated with a slight reduction in food intake in humans [8], [9] and baboons [10]. While these studies suggest an important role for circulating glucose action within the CNS to influence food intake, it remains unknown if physiological fluctuations of glucose availability, per se, regulate food intake. An alternative possibility is that glucose, in and of itself, is instead permissive for other signals to suppress food intake.

Glucagon-like peptide-1 (GLP-1), a neuropeptide that is secreted postprandially from the gut [11], augments insulin release [12], [13], and this effect is dependent upon the availability of glucose [14]–[16]. This has important therapeutic implications for GLP-1-based therapies since it means that these drugs are less likely than other insulin-secretagogues to cause hypoglycemia. GLP-1 is also made in the brain, and administration of GLP-1 into i3vt [17]–[21], or directly into the paraventricular [22], [23] dorsomedial, and ventromedial hypothalamus [24], and the brainstem [25] all significantly reduce food intake. Analogous to GLP-1 action at the β-cell, there is some evidence that CNS actions of GLP-1 are also nutrient-dependent. Prolonged fasting blunts the anorexic effects of both peripheral GLP-1 and exendin-4, a long-acting GLP-1 receptor agonist [26], and recently, Hayes et al. [27] reported that the anorexic effect of exendin-4, is mediated by inhibiting activity of the fuel sensor, AMPK, within the hindbrain. Thus, multiple points of evidence suggest that fuel sensing within the CNS is important for CNS GLP-1 action. In this study, we tested the hypothesis that glucose availability within the hypothalamus influences activation of the CNS GLP-1r specifically by GLP-1.

## Materials and Methods

### Animal Preparation

Male (∼300 g) Long Evans rats were purchased from Harlan (Indianapolis, IN), and glucokinase heterozygote mice were purchased from Jackson Laboratories (Bar Harbor, ME). Animals were singly-housed according in the University of Cincinnati Laboratory Animals for Medical Science Facility at the Metabolic Diseases Institute under controlled conditions (12∶12 light-dark cycle, 50–60% humidity, 25°C) with free access to standard rodent chow diet and water except where noted. All procedures for animal use were approved by The University of Cincinnati Institutional Animal Care and Use Committee and the principles of laboratory animal care as stated by the NIH were followed.

### Surgeries

Multiple cohorts of animals were studied. In some cohorts, rats had a stainless steel cannula placed in the i3vt (22-ga, 11 mm) using stereotaxic (David Kopf Instruments, Tujunga, California) coordinates as determined by the atlas of Paxinos and Watson [28]. Cannula placement was verified in rats drinking ≥5 ml of water in the hour following injection of angiotensin II (10 nmol/1 µl) into the CNS. A small percentage (<1%) of animals who did not drink in response to angiotensin II were excluded from all studies.

### Effects of i3vt GLP-1 in fed vs. Fasted Animals

Animals (n = 8/group) were fed *ad lib* or had food removed 2 h after the onset of dark in these sets of studies. The next day, prior to the onset of dark, fed or 22-h fasted animals received 3 µg of GLP-1 (American Peptide, Sunnyvale, CA), or 2 µl of saline vehicle directly into the i3vt. To test the specificity of fasting-induced changes on GLP-1 action, in another study, melanotan II, a melanocortin receptor agonist (MTII; Phoenix Pharmaceuticals, Mountain View, CA; 0.1 µg) or saline (2 µl) was injected into the i3vt 4 h prior to lights out in fed or 22-h fasted animals. In both sets of studies, food was returned and intake was measured at 1, 3 and 24 h after the onset of the dark cycle.

### Effects of Leptin on i3vt GLP-1 Function

In another cohort of animals, we assessed whether leptin could restore GLP-1-induced anorexia in fasted animals. I3vt leptin (Calbiochem, San Diego, CA; 3 µg) or saline (1 µl) was injected prior to i3vt GLP-1 (3 µg) or saline (2 µl) in 22-h fasted animals (n = 15/group). In another study (n = 12/group), i3vt leptin (3 µg) or saline (3 µl) was injected prior to intraperitoneal (ip) exendin-4 (1 µg/kg) or water vehicle. We used doses of GLP-1 and leptin that alone had small effects on food intake. This methodological design is important because it allows us to determine the potential *additive* or *non-additive* effect of GLP-1 and leptin (or other double drug manipulations below) on food intake [29].

### Effects of Glucose Availability on GLP-1 Function

2-deoxyglucose (2DG, Sigma-Aldrich, St. Louis, MO) is a glucose analog that inhibits the enzyme phosphohexose isomerase, and thus blocks the first reaction in glycolysis (conversion of glucose-6-phosphate to fructose-6-phosphate). 2DG has potent glucopenic actions within the CNS and significantly increases food intake. We first determined a subthreshold dose of i3vt 2DG that did not increase food intake by administering saline, 1, 3, or 10 µg of 2DG into the i3vt. This allowed us to use a dose of 2DG that is specifically blocks rather than simply *overpowers* GLP-1 action. One week after this study, and one hour prior to lights out, a subthreshold dose of 2DG (1 µg) or saline was injected into the i3vt immediately prior to i3vt GLP-1 (3 µg/2 µl) or saline (2 µl) in ad lib fed animals and food intake was measured (n = 8/group) as above.

We also tested whether AMPK activation, another cellular signal of nutrient deprivation, could block GLP-1 action. The protocol was similar as with 2DG except with i3vt synthetic cerebral spinal fluid as the vehicle or i3vt 5-amino imidazole-4-carboxamide-riboside (AICAR; 300 µg/3 µl; Acros Organics, Geel, Belgium, prepared as described in [30]) was administered to ad lib fed animals (n = 8/group) and food intake was measured as described above. The dose of AICAR was chosen because it was previously shown to have a subthreshold effect on food intake [27].

We then tested whether increasing CNS glucose availability could potentiate GLP-1 function in fasted animals. In two sets of studies, 2 different doses of glucose (0.6 µg; n = 7/group and 540 µg; n = 12/group) or saline (2 µl) was injected i3vt immediately prior to GLP-1 (3 µg) or saline (2 µl) in 22-h fasted rats and food intake at the onset of dark was assessed. We administered i3vt glucose first at a dose of 0.6 µg because preliminary data from our group suggested that this was a subthreshold dose (unpublished data). We subsequently 540 µg of glucose which is a higher subthreshold dose that have been used previously [5]. In another set of studies IP (1.5 g/kg) or oral gavage of glucose (2 g/kg) (vs. saline and water, respectively) was administered after i3vt GLP-1 (3 µg) or saline (2 µl) and food intake after the onset of dark was assessed. These doses of glucose were chosen as they are typical doses used for IP and oral GTTs and because we have found that they both significantly elevate blood glucose and insulin levels in rats [31], [32].

### Glucokinase +/− Mice

To examine the role of glucokinase (GK), an important CNS glucose sensor [33] we studied wild-type and GK-heterozygote knockout (GK+/−; Stock No. 003264, Jackson Laboratories, Bar Harbor, ME) mice. After a 4-h fast and on different occasions, IP injections of saline (0.3 ml), GLP-1 (500 µg), or a dipetidyl peptide-4 inhibitor, vildagliptin (150 µg; provided by Norvartis Pharmaceuticals, East Hanover, NJ), which extends the half-life of GLP-1, or GLP-1 (500 µg)+Vildagliptin (150 µg) were administered immediately before lights out. Food was weighed at 1, 2, 4, and 24 h after lights-out to assess food intake. On another occasion, food was removed 4 h prior to lights out and an IP injection of saline or exendin-4 (100 µg/kg) was administered and food-intake was assessed as described above.

### Statistical Analysis

The data were analyzed using mixed-model ANOVAs with a Tukey’s post-hoc analysis where appropriate. Statistical significance was set at p<0.05 for all analyses. Data are presented as mean ± SE.

## Results

### Effect of Nutrient Status on i3vt GLP-1-induced Anorexia

**Figure 1:**
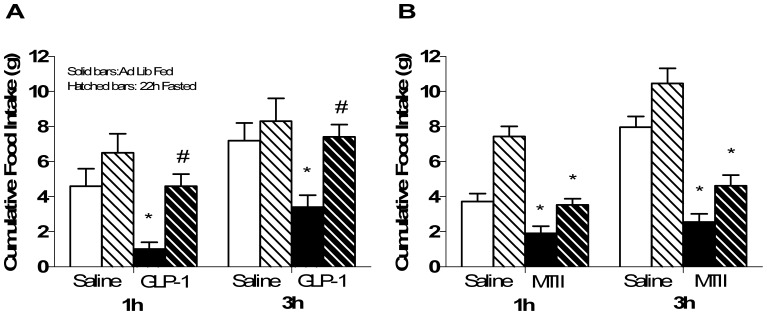
Food intake after i3vt GLP-1 or MTII or saline in fed or fasted animals. a) i3vt GLP-1 suppressed food intake in ad lib (solid bars) fed but not 22 h fasted(hatched bars) animals. *p<0.05 i3vt GLP-1 vs. saline within the ad lib animals; ^#^p<0.05 i3vt GLP-1 fasted vs. ad lib fed animals. b) 1 and 3 h food intake was equally suppressed after i3vt melanotan II (MTII) in ad lib fed and fasted for 22 h. *p<0.05 vs. ICV saline in both fed and fasted groups.

**Figure 2:**
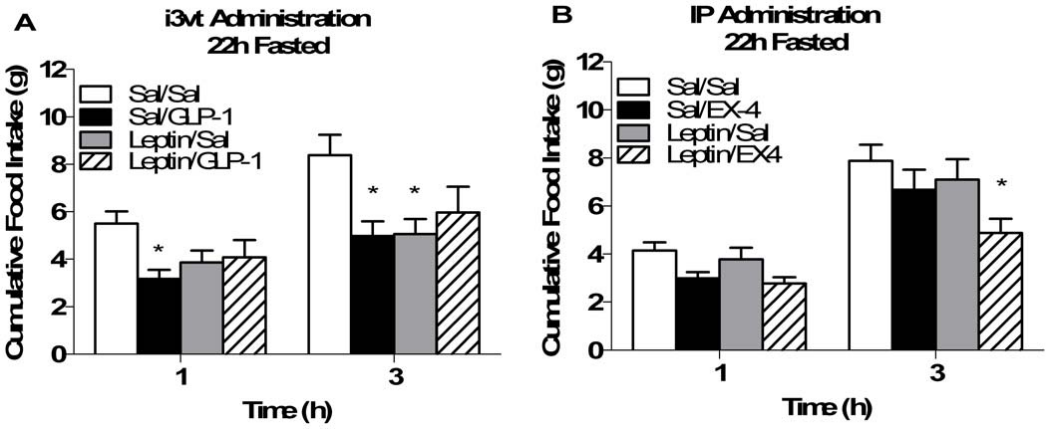
Interaction of the GLP-1 and leptin systems on food intake after fasting. a) 1 and 3 h food intake was suppressed in animals given i3vt GLP-1 and 3h food intake was suppressed by i3vt leptin (*p<0.05 vs. i3vt saline). There was no additive effect of giving GLP-1+ leptin on suppression of food intake in 22h fasted animals. White bar, i3vt saline+i3vt saline; black bar, i3vt saline+i3vt GLP-1; gray bar, i3vt leptin+ i3vt saline; hatched bar, i3vt leptin+i3vt GLP-1. b) 3 h food intake was suppressed by combined IP injections of EX4 and leptin. *p<0.05 in EX4+leptin vs. all other groups at the 3 h time point. White bar, i3vt saline+IP saline; black bar, i3vt saline+IP exendin-4; gray bar, i3vt leptin+IP saline; hatched bar, i3vt leptin+IP exendin-4.

I3vt injected GLP-1 significantly reduced food intake in ad lib-fed animals at 1 and 3 h after lights out vs. saline. In contrast, 22-h-fasted animals had no significant reduction in food intake over time and had significantly greater food intake compared to ad lib-fed animals given GLP-1 (p<0.05 GLP-1 fed vs. all other groups; Figure 1a). In contrast, this same degree of fasting had no affect on the ability of MTII, another anorexic agent, to reduce food intake 3 h after lights out (p<0.05 MTII vs. saline regardless of feeding status; Figure 1b) suggesting that being fasted does not block all drugs that cause anorexia. I3vt leptin prior to i3vt GLP-1 did not potentiate the ability of GLP-1 to reduce food intake under fasted conditions (Figure 2a). Three h after the injections, both leptin and GLP-1 caused significantly less cumulative food intake compared to saline, but there was no additive effect of giving leptin and GLP-1 together (p>0.05 i3vt leptin+GLP-1 vs. each drug alone; Figure 2a). Conversely, we were able to replicate findings [26] that i3vt leptin potentiated the ability of IP exendin 4, to reduce food intake (p<0.05 i3vt leptin+IP exendin 4 vs. all other groups; Figure 2b).

### Glucose Dependence of GLP-1

**Figure 3:**
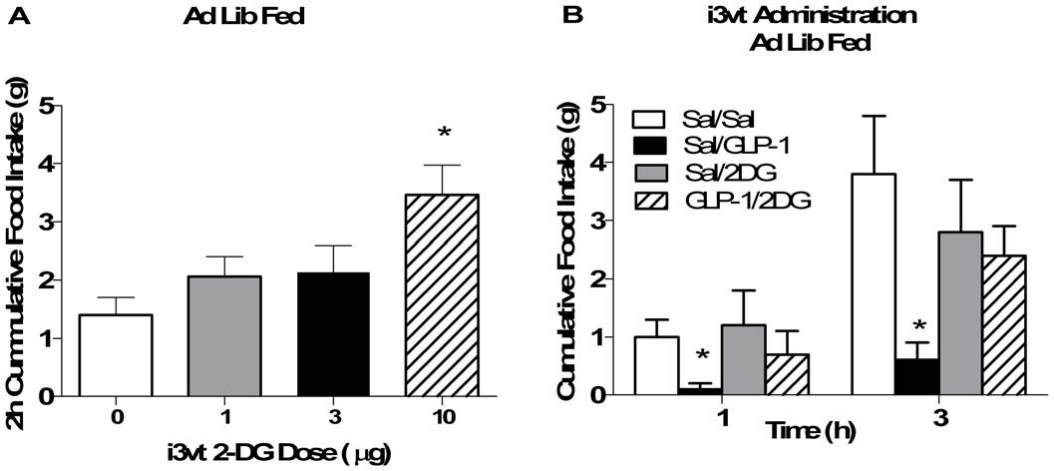
The impact of blocking CNS glucose metabolism on the anorectic ability of GLP-1 in fed animals. a) The subthreshold orexigenic dose was assessed in ad lib fed animals after saline (0; white bar), 1 (gray bar), 3 (black bar) or 10 (hatched bar) µg of i3vt 2DG. Food intake was significantly increased by the 10 µg dose over all other doses. The 1 and 3 µg doses had no significant effect on food intake. *p<0.05 i3vt 2DG vs. all other groups. b) Food intake was suppressed after i3vt GLP-1 but not after i3vt GLP-1+2DG in fed animals (*p<0.05 i3vt GLP-1+2DG vs. all other groups). White bar, i3vt saline+ i3vt saline; black bar, i3vt GLP-1+ i3vt saline; gray bar, i3vt saline+ i3vt 2DG; hatched bar, i3vt GLP-1+ i3vt 2DG.

**Figure 4:**
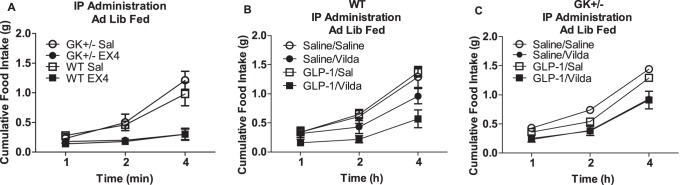
The impact of glucokinase on the anorexic effect of GLP-1 in fed animals. a) Food intake was equally suppressed in WT (squares) and GK+/− (circles) mice after IP injection of EX4 (solid symbols) vs. saline (open symbols; *p<0.05 at 4 h after injection). Food intake was also equally suppressed at 4 h by IP GLP-1+vildagliptin in WT (b) and GK+/− (c) mice (*p<0.05 at 4 h post-injection). For b) and c), open circle, IP saline+IP saline; closed circle, IP saline+IP vildagliptin; open square, IP GLP-1+IP saline; closed square, IP GLP-1+IP vildagliptin.

**Figure 5:**
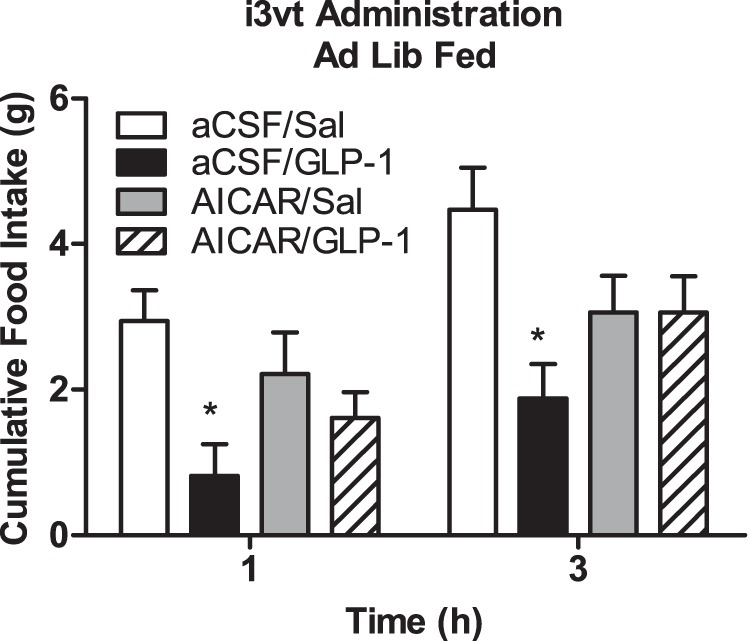
The impact of AICAR on GLP-1-induced anorexia. Animals given CSF+GLP-1 had significantly reduced food intake at 1 and 3 h compared to all other groups (*p<0.05) and AICAR blocked this effect. Open bar, i3vt artificial cerebral spinal fluid+ i3vt saline; closed bar, i3vt artificial cerebral spinal fluid+i3vt GLP-1; gray bar, i3vt AICAR+ i3vt saline; hatched bar, i3vt AICAR+i3vt GLP-1.

To determine if the ability of central GLP-1 to reduce food intake is dependent on glucose availability, we administered i3vt 2DG prior to GLP-1. At a dose that did not independently increase food intake (1 µg), i3vt 2DG blocked GLP-1-induced suppression of food intake (p<0.05 i3vt 2DG+GLP-1 vs. all other groups; Figure 3). To determine if glucokinase, an important neuronal glucose sensor [34], is a mediator of GLP-1 action, we administered IP GLP-1 plus vildagliptin, or IP exendin-4, a long-acting GLP-1 agonist, to GK-heterozygote knockout and littermate control mice. In our preliminary studies, we do not see an impact of an IP injection of GLP-1 alone to reduce food intake (data not shown). Therefore, as a preliminary study we used exendin-4 to determine if glucokinase deficiency could block the effect of exendin-4. Because exendin-4 is a pharmacological agonist for GLP-1, we also injected GLP-1 with the DPP4 inhibitor to determine if glucokinase deficiency could impair endogenously-induced GLP-1R signaling. The glucokinase-heterozygote mice responded similarly to the anorexic effects of IP GLP-1 plus vildagliptin, and to IP exendin-4, compared to wild-type animals (p>0.05 for drugXgenotype interaction; Figure 4a-c). Interestingly, activation of CNS AMPK (another fuel-sensor that is activated when fuel sources are low) with i3vt AICAR did significantly block the ability of i3vt GLP-1 to inhibit food intake (p<0.05 GLP-1 vs. AICAR and saline; Figure 5).

**Figure 6:**
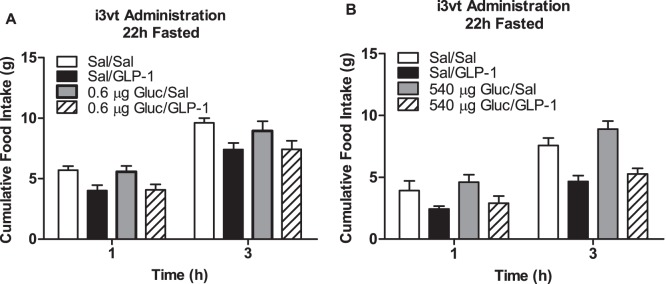
The ability of i3vt glucose to potentiate the anorexic effect of GLP-1 in fasted animals. Food intake after i3vt glucose at doses of a) 0.6 µg or b) 540 µg or saline plus GLP-1 or saline in 22 h fasted animals. With both doses of glucose, there was a main effect of drug with animals given GLP-1, regardless of glucose dose, having suppressed food intake (p<0.05; main effect of group). Open bar, i3vt saline+ i3vt saline; closed bar, i3vt saline+i3vt GLP-1; gray bar, i3vt glucose+ i3vt saline; hatched bar, i3vt glucose+i3vt GLP-1.

**Figure 7:**
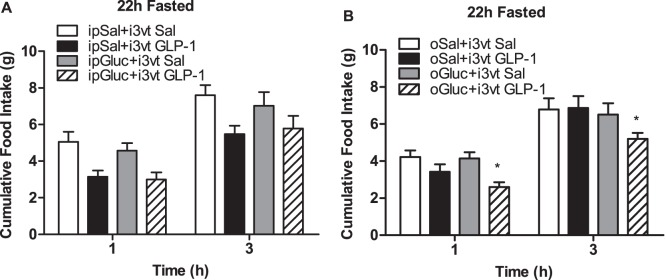
The ability of peripherally administered glucose to potentiate the anorexic effect of GLP-1 in fasted animals. Food intake after a) IP or b) oral glucose prior to an i3vt injection of GLP-1 or saline in fasted rats. Oral, but not IP glucose suppressed food intake after i3vt GLP-1 (*p>0.05 vs. ICV GLP-1+glucose, independent of time). Open bar, IP (a) or oral (b) saline+ i3vt saline; closed bar, IP (a) or oral (b) saline+i3vt GLP-1; gray bar, IP (a) or oral (b) glucose+ i3vt saline; hatched bar, IP (a) or oral (b) glucose+i3vt GLP-1.

To determine if providing glucose could restore central GLP-1 action in fasted animals, we administered i3vt glucose at a dose of 0.6 µg (based on preliminary data) and at 540 µg, a dose previously shown to suppress food intake [5]. Importantly, neither dose acted independently to reduce food intake, however, they also did not act synergistically with GLP-1 to reduce food intake (p>0.05; Figure 6a and b, respectively). We then systemically administered IP glucose (1.5 g/kg), which also did not independently or synergistically with GLP-1 reduce food intake (p>0.05; Figure 7a). However, when given together, oral glucose and GLP-1 significantly reduced food intake compared to all other groups independent of time in fasted rats (p<0.05 GLP-1+glucose vs. all other groups; Figure 7b).

## Discussion

GLP-1 has a wide array of physiological actions that depend on the specific neurons in the CNS where its receptors are located. One of these actions within the hypothalamus is a potent reduction of food intake. Our data add to this by illustrating that hypothalamic and peripheral fuel-sensing mechanisms are important in mediating GLP-1-induced reductions in food intake. In the current study, we demonstrate that activation of hypothalamic GLP-1r, specifically by GLP-1, is modulated by hypothalamic AMPK as well as by GI glucose-sensing pathways.

We found that MTII had similar anorexic effects whether animals were fed or fasted suggesting that fasting, per se, does not block all anorexic peptides. While it’s possible that MTII is simply more potent than GLP-1, we do not believe this is the case since we used a moderate dose of MTII [35] and because the dose of GLP-1 and MTII were equally anorexic at 1 h and 3 h after administration in fed animals. Nevertheless, in order to gain greater understanding of the integration of nutrient status to regulation of food intake, it would be interesting to examine the anorectic action of other peptides under both fed and fasting conditions.

Our current data demonstrate that CNS GLP-1r activation via direct i3vt administration of GLP-1 is not potentiated by a co-injection of leptin suggesting a lack of additivity of these two receptor populations on regulating food intake. GLP-1r have been found to be co-localization to leptin receptors within the hindbrain [36], and viral [37] or genetic [36] suppression of leptin receptor expression within the NTS/AP increases fat mass by increasing food intake, Further, leptin has been found to potentiate the action of peripheral GLP-1 and exendin-4 under fasting conditions [26]. Altogether, these findings could indicate that there are regionally specific interactions of the GLP-1r and the leptin receptor and our data suggest that the hypothalamic region is not one of these interactive areas. However, it is also important to note that our studies were done with i3vt GLP-1 rather than exendin-4. Central administration of exendin-4 is a 100 fold more potent and even when administered at doses that have comparable anorexic effects, exendin-4 is resistant to two different GLP-1r antagonists [30]. Thus, there could be differences in the relationship between nutrient sensing and GLP-1 vs. exendin-4 when the drugs are administered via CNS (our study) vs. peripherally [26].

While 2DG, a glucose analog that inhibits the first reaction in glycolysis, was able to block the ability of i3vt GLP-1 to suppress food intake, we found that GLP-1 and exendin-4 suppressed food intake equally in wild type and glucokinase-heterozygote mice suggesting that glucokinase is not the glucose-sensing between feeding state and GLP-1r activation. We cannot fully rule out the impact of glucokinase since it is possible that the glucokinase heterozygote mice had sufficient amount and/or function of neuronal glucokinase to allow the GLP-1 system to function normally. However, 2DG could also impact GLP-1r signaling via its ability to activate AMPK, another important CNS fuel sensor [31]. When we gave i3vt AICAR, an AMPK activator, we were also able to block the anorectic action of GLP-1. Thus, our data point to the possibility that 2DG blocked GLP-1 action via downstream activation of AMPK. One potential interpretation of these data is that during fasting, AMPK is activated, and this activation consequently blocks hypothalamic GLP-1 action. When nutrients are available (oral glucose for example), AMPK is de-activated, restoring GLP-1 function. In support of this, fourth cerebroventricular administration of AICAR has also been shown to block exendin-4 action on the GLP-1r [27] suggesting that whether it’s via AMPK or off target effects, AICAR has consistent affects on GLP-1r activation.

While blocking glucose or fuel availability to the CNS with 2DG or AICAR blocked the anorectic action of GLP-1, direct CNS administration of glucose did not potentiate GLP-1 action. Interestingly, oral, but not IP glucose administration did potentiate the ability of GLP-1 to reduce food intake in fasted conditions. This suggests that gut-to-brain communication is important for hypothalamic GLP-1 signaling. Given that retrograde intracarotid glucose administration increases neuronal activity as measured by c-Fos immunoreactivity in several regions of the hypothalamus [32] whereas oral glucose decreases neuronal activity in some of these same regions [33], it is possible that the CNS processes glucose signals differentially depending on the route of administration. Certainly, oral glucose initiates myriad of signals to the CNS which in concert contribute to a differential response. One of the many possibilities is that oral, but not IP glucose, will stimulate the release of GLP-1 from L-cells and this in turn potentiates hypothalamic GLP-1 action. The interaction of peripherally-released GLP-1 and brain-released GLP-1 is not clearly understood. Although GLP-1 has been found to cross the blood brain barrier and the median eminence may allow access of blood derived GLP-1 to the hypothalamus, the rapid half-life of GLP-1 (<2 min) in the circulation makes endocrine action on the CNS unlikely. However, a neural route of communication between the peripheral and central GLP-1 systems has been suggested [34]–[36], and subdiaphragmatic vagotomy has been found to blunt the suppressive effects of long-acting GLP-1r agonists on food intake [37]. In support of this, the anorectic effect of gastric distention is blocked by injection of a GLP-1r antagonist administered directly into the NTS [38]. Thus, it is possible that gastrointestinal-to-CNS communication is able to potentiate hypothalamic GLP-1 action.

In summary, while GLP-1 action specifically within the CNS does seem to be fuel dependent, the peripheral and CNS systems likely contain redundant fuel sensing mechanisms including within the GI tract in order to integrate nutrient status with regulation of food intake. While AMPK, a general fuel sensor, is important within the CNS, direct GI glucose sensing is also important for GLP-1 action. GLP-1 has a wide array of physiological functions not limited to improving postprandial glucose homeostasis but including activation of the hypothalamic pituitary-adrenal-axis and the sympathetic nervous system. Distinguishing among these different fuel-sensing mechanisms may link specific actions of GLP-1 to these various functions.

## References

[1] Mayer J (1955) Regulation of energy intake and the body weight: the glucostatic theory and the lipostatic hypothesis. Annals of the New York Academy of sciences 63: 15–43. Available: http://onlinelibrary.wiley.com/doi/10.1111/j.1749-6632.1955.tb36543.x/abstract. Accessed 1 December 2011. 10.1111/j.1749-6632.1955.tb36543.x 13249313

[2] Sanders NM, Ritter S (2001) Hindbrain glucoreceptive sites exhibit impaired responsiveness to gluocoprivation following systemic 2-deoxy-D-glucose (2DG)-induced glucoprivation or dexamethasone (DEX). Diabetes 50: A53. 10.2337/diabetes.50.12.2831 11723067

[3] Hudson B, Ritter S (2004) Hindbrain catecholamine neurons mediate consummatory responses to glucoprivation. Physiol Behav 82: 241–250 Available: http://www.ncbi.nlm.nih.gov/entrez/query.fcgi?cmd=Retrieve&db=PubMed&dopt=Citation&list_uids=15276785. 15276785 10.1016/j.physbeh.2004.03.032

[4] Ritter RC, Slusser P (1980) 5-Thio-D-glucose causes increased feeding and hyperglycemia in the rat. Am J Physiol 238: E141–4 Available: http://www.ncbi.nlm.nih.gov/htbin-post/Entrez/query?db=m&form=6&dopt=r&uid=7361889. 7361889 10.1152/ajpendo.1980.238.2.E141

[5] Kurata K, Fujimoto K, Sakata T, Etou H, Fukagawa K (1986) D-glucose suppression of eating after intra-third ventricle infusion in rat. Physiology & Behavior 37: 615 Available: http://www.sciencedirect.com/science/article/pii/0031938486902957. 3749325 10.1016/0031-9384(86)90295-7

[6] Davis JD, Wirtshafter D, Asin KE, Brief D (1981) Sustained intracerebroventricular infusion of brain fuels reduces body weight and food intake in rats. Science 212: 81–83 Available: http://www.ncbi.nlm.nih.gov/entrez/query.fcgi?cmd=Retrieve&db=PubMed&dopt=Citation&list_uids=7193909. 7193909 10.1126/science.7193909

[7] Gilbert M, Magnan C, Turban S, André J, Guerre-Millo M (2003) Leptin Receptor-Deficient Obese Zucker Rats Reduce Their Food Intake in Response to a Systemic Supply of Calories From Glucose. Diabetes 52: 277–282 Available: http://diabetes.diabetesjournals.org/content/52/2/277.abstract. 12540597 10.2337/diabetes.52.2.277

[8] Russell AW, Horowitz M, Ritz M, MacIntosh C, Fraser R, et al (2001) The effect of acute hyperglycaemia on appetite and food intake in Type 1 diabetes mellitus. Diabetic Medicine 18: 718 Available: http://dx.doi.org/10.1046/j.1464-5491.2001.00545.x. 11606169 10.1046/j.1464-5491.2001.00545.x

[9] Gielkens HAJ, Verkijk M, Lam WF, Lamers CBHW, Masclee AAM (1998) Effects of hyperglycemia and hyperinsulinemia on satiety in humans. Metabolism 47: 321 Available: http://www.sciencedirect.com/science/article/pii/S0026049598902645. 9500570 10.1016/s0026-0495(98)90264-5

[10] Woods SC, Stein LJ, McKay LD, Porte Jr D (1984) Suppression of food intake by intravenous nutrients and insulin in the baboon. Am J Physiol 247: R393–R401. 6380317 10.1152/ajpregu.1984.247.2.R393

[11] D’Alessio DA, Thirlby R, Laschansky EC, Zebroski H, Ensinck JW (1993) Response of GLP-1 to nutrients in humans. Digestion 54: 377–379.

[12] Kreymann B, Ghatei MA, Williams G, Bloom SR (1987) Glucagon-like peptide-1 7–36: A physiological incretin in man. Lancet 2: 1300–1303. 2890903 10.1016/s0140-6736(87)91194-9

[13] Mojsov S, Weir GC, Habener J (1987) Insulinotropin: Glucagon-like peptide 1 (7–37) co- encoded in the glucagon gene is a potent stimulator of insulin release in the perfused rat pancreas. Journal of Clinical Investigation 79: 616–619. 3543057 10.1172/JCI112855 PMC424143

[14] Weir GC, Mojsov S, Hendrick GK, Habener JF (1989) Glucagonlike peptide I (7–37) actions on endocrine pancreas. Diabetes 38: 338–342 Available: http://www.ncbi.nlm.nih.gov/entrez/query.fcgi?cmd=Retrieve&db=PubMed&dopt=Citation&list_uids=2645190. 2645190 10.2337/diab.38.3.338

[15] Meier JJ, Gallwitz B, Schmidt WE, Nauck MA (2002) Glucagon-like peptide 1 as a regulator of food intake and body weight: therapeutic perspectives. European Journal of Pharmacology 440: 269–279. 12007541 10.1016/s0014-2999(02)01434-6

[16] Qualmann C, Nauck MA, Holst JJ, Orskov C, Creutzfeldt W (1995) Glucagon-like peptide 1 (7–36 amide) secretion in response to luminal sucrose from the upper and lower gut. A study using alpha-glucosidase inhibition (acarbose). Scand J Gastroenterol 30: 892–896. 8578189 10.3109/00365529509101597

[17] Turton MD, O’Shea D, Gunn I, Beak SA, Edwards CMB, et al (1996) A role for glucagon-like peptide-1 in the central regulation of feeding. Nature 379: 69–72. 8538742 10.1038/379069a0

[18] Tang-Christensen M, Vrang N, Larsen PJ (1998) Glucagon-like peptide 1(7–36) amide’s central inhibition of feeding and peripheral inhibition of drinking are abolished by neonatal monosodium glutamate treatment. Diabetes 47: 530–537. 9568683 10.2337/diabetes.47.4.530

[19] van Dijk G, Thiele TE, Seeley RJ, Woods SC, Bernstein IL (1997) Glucagon-like peptide-1 and satiety [letter; comment]. Nature 385: 214. 9000071 10.1038/385214a0

[20] van Dijk G, Thiele TE, Donahey JCK, Campfield LA, Smith FJ, et al (1996) Central infusion of leptin and GLP-1 (7–36) amide differentially stimulate c-Fos-like immunoreactivity in the rat brain. American Journal of Physiology 271: R1096–R1100. 8898006 10.1152/ajpregu.1996.271.4.R1096

[21] Tang-Christensen M, Larsen PJ, Goke R, Fink-Jensen A, Jessop DS, et al (1996) Central administration of GLP-1-(7–36) amide inhibits food and water intake in rats. American Journal of Physiology 271: R848–856. 8897973 10.1152/ajpregu.1996.271.4.R848

[22] Kinzig KP, D’Alessio DA, Seeley RJ (2002) The diverse roles of specific GLP-1 receptors in the control of food intake and the response to visceral illness. J Neurosci 22: 10470–10476 Available: http://www.ncbi.nlm.nih.gov/entrez/query.fcgi?cmd=Retrieve&db=PubMed&dopt=Citation&list_uids=12451146. 12451146 10.1523/JNEUROSCI.22-23-10470.2002 PMC6758755

[23] McMahon LR, Wellman PJ (1998) PVN infusion of GLP-1-(7–36) amide suppresses feeding but does not induce aversion or alter locomotion in rats. Am J Physiol 274: R23–9. 9458894 10.1152/ajpregu.1998.274.1.R23

[24] Schick RR, Zimmermann JP, vorm Walde T, Schusdziarra V (2003) Peptides that regulate food intake: glucagon-like peptide 1-(7–36) amide acts at lateral and medial hypothalamic sites to suppress feeding in rats. Am J Physiol Regul Integr Comp Physiol 284: R1427–35 Available: http://www.ncbi.nlm.nih.gov/entrez/query.fcgi?cmd=Retrieve&db=PubMed&dopt=Citation&list_uids=12776726. 12776726 10.1152/ajpregu.00479.2002

[25] Grill HJ, Carmody JS, Amanda Sadacca L, Williams DL, Kaplan JM (2004) Attenuation of lipopolysaccharide anorexia by antagonism of caudal brain stem but not forebrain GLP-1-R. Am J Physiol Regul Integr Comp Physiol 287: R1190–3 Available: http://www.ncbi.nlm.nih.gov/entrez/query.fcgi?cmd=Retrieve&db=PubMed&dopt=Citation&list_uids=15231492. 15231492 10.1152/ajpregu.00163.2004

[26] Williams DL, Baskin DG, Schwartz MW (2006) Leptin regulation of the anorexic response to glucagon-like peptide-1 receptor stimulation. Diabetes 55: 3387–3393 Available: http://www.ncbi.nlm.nih.gov/entrez/query.fcgi?cmd=Retrieve&db=PubMed&dopt=Citation&list_uids=17130484. 17130484 10.2337/db06-0558

[27] Hayes MR, Leichner TM, Zhao S, Lee GS, Chowansky A, et al (2011) Intracellular signals mediating the food intake-suppressive effects of hindbrain glucagon-like peptide-1 receptor activation. Cell Metab 13: 320–330 Available: http://www.ncbi.nlm.nih.gov/entrez/query.fcgi?cmd=Retrieve&db=PubMed&dopt=Citation&list_uids=21356521. 21356521 10.1016/j.cmet.2011.02.001 PMC3108145

[28] Paxinos G, Watson C (1986) The Rat Brain in Stereotaxic Coordinates. 2nd ed. San Diego: Academic Press, Inc.

[29] Seeley RJ, Moran TH (2002) Principles for interpreting interactions among the multiple systems that influence food intake. American Journal of Physiology 283: R46–53 Available: http://www.ncbi.nlm.nih.gov/htbin-post/Entrez/query?db=m&form=6&dopt=r&uid=12069929. 12069929 10.1152/ajpregu.00021.2002

[30] Barrera JG, D’Alessio DA, Drucker DJ, Woods SC, Seeley RJ (2009) Differences in the central anorectic effects of GLP-1 and exendin-4 in rats. Diabetes. Available: http://www.ncbi.nlm.nih.gov/entrez/query.fcgi?cmd=Retrieve&db=PubMed&dopt=Citation&list_uids=19741167. 10.2337/db09-0281 PMC2780868 19741167

[31] Minokoshi Y, Alquier T, Furukawa N, Kim YB, Lee A, et al (2004) AMP-kinase regulates food intake by responding to hormonal and nutrient signals in the hypothalamus. Nature 428: 569–574 Available: http://www.ncbi.nlm.nih.gov/entrez/query.fcgi?cmd=Retrieve&db=PubMed&dopt=Citation&list_uids=15058305. 15058305 10.1038/nature02440

[32] Dunn-Meynell AA, Govek E, Levin BE (1997) Intracarotid glucose selectively increases Fos-like immunoreactivity in paraventricular, ventromedial and dorsomedial nuclei neurons. Brain Res 748: 100–106. 9067450 10.1016/s0006-8993(96)01280-2

[33] Knauf C, Cani PD, Kim DH, Iglesias MA, Chabo C, et al (2008) Role of central nervous system glucagon-like Peptide-1 receptors in enteric glucose sensing. Diabetes 57: 2603–2612 Available: http://www.ncbi.nlm.nih.gov/entrez/query.fcgi?cmd=Retrieve&db=PubMed&dopt=Citation&list_uids=18519802. 18519802 10.2337/db07-1788 PMC2551668

[34] Vahl TP, Tauchi M, Durler TS, Elfers EE, Fernandes TM, et al (2007) Glucagon-like peptide-1 (GLP-1) receptors expressed on nerve terminals in the portal vein mediate the effects of endogenous GLP-1 on glucose tolerance in rats. Endocrinol 148: 4965–4973 Available: http://www.ncbi.nlm.nih.gov/entrez/query.fcgi?cmd=Retrieve&db=PubMed&dopt=Citation&list_uids=17584962. 10.1210/en.2006-0153 17584962

[35] Washington MC, Raboin SJ, Thompson W, Larsen CJ, Sayegh AI (2010) Exenatide reduces food intake and activates the enteric nervous system of the gastrointestinal tract and the dorsal vagal complex of the hindbrain in the rat by a GLP-1 receptor. Brain Res 1344: 124–133 Available: http://www.ncbi.nlm.nih.gov/entrez/query.fcgi?cmd=Retrieve&db=PubMed&dopt=Citation&list_uids=20452329. 20452329 10.1016/j.brainres.2010.05.002

[36] Amato A, Cinci L, Rotondo A, Serio R, Faussone-Pellegrini MS, et al (2010) Peripheral motor action of glucagon-like peptide-1 through enteric neuronal receptors. Neurogastroenterol Motil 22: 664–e203 Available: http://www.ncbi.nlm.nih.gov/entrez/query.fcgi?cmd=Retrieve&db=PubMed&dopt=Citation&list_uids=20158614. 20158614 10.1111/j.1365-2982.2010.01476.x

[37] Kanoski SE, Fortin SM, Arnold M, Grill HJ, Hayes MR (2011) Peripheral and Central GLP-1 Receptor Populations Mediate the Anorectic Effects of Peripherally Administered GLP-1 Receptor Agonists, Liraglutide and Exendin-4. Endocrinology 152: 3103–3112 Available: http://endo.endojournals.org/content/152/8/3103.abstract. 21693680 10.1210/en.2011-0174 PMC3138234

[38] Hayes MR, Bradley L, Grill HJ (2009) Endogenous Hindbrain Glucagon-Like Peptide-1 Receptor Activation Contributes to the Control of Food Intake by Mediating Gastric Satiation Signaling. Endocrinology 150: 2654–2659 Available: http://endo.endojournals.org/cgi/content/abstract/150/6/2654. 19264875 10.1210/en.2008-1479 PMC2689794

